# Insight in Individuals With First-episode Psychosis: Correlates With Symptoms, Neurocognition and Psychosocial Functioning Over Acute and Stable Phases

**DOI:** 10.62641/aep.v53i4.1925

**Published:** 2025-08-05

**Authors:** Mar Mamano-Grande, Paola Punsoda-Puche, Victoria Espinosa, Judith Usall, Ana Barajas, Iris Baños, Bernardo Sánchez, Montse Dolz, Susana Ochoa

**Affiliations:** ^1^Parc Sanitari Sant Joan de Déu, Sant Boi de Llobregat, 08830 Barcelona, Spain; ^2^Fundació Sant Joan de Déu, Esplugues de Llobregat, 08950 Barcelona, Spain; ^3^Institut de Recerca Sant Joan de Déu (IRSJD), Esplugues de Llobregat, 08950 Barcelona, Spain; ^4^Etiopatogènia i Tractament dels Trastorns Mentals Greus (MERITT), Institut de Recerca Sant Joan de Déu, Parc Sanitari Sant Joan de Déu, Sant Boi de Llobregat, 08830 Barcelona, Spain; ^5^Centro de Investigación Biomédica en Red de Salud Mental, Instituto de Salud Carlos III, 28029 Madrid, Spain; ^6^Departament de Psicologia Clínica i de la Salut, Facultat de Psicologia, Universitat Autònoma de Barcelona, Bellaterra, Cerdanyola del Vallès, 08193 Barcelona, Spain; ^7^Serra Húnter Fellow, Generalitat de Catalunya, 08002 Barcelona, Spain; ^8^Centre d'Higiene Mental Les Corts, 08029 Barcelona, Spain; ^9^Hospital Mutua de Terrassa, Terrassa, 08221 Barcelona, Spain; ^10^Hospital Sant Joan de Déu de Barcelona, Esplugues de Llobregat, 08906 Barcelona, Spain

**Keywords:** insight, first-episode psychosis, symptoms, neuropsychology, psychosocial functioning

## Abstract

**Background:**

Poor insight is prevalent in individuals with first-episode psychosis (FEP) and is associated with unfavorable outcomes. Despite distinctions in insight characteristics between FEP and established schizophrenia, further research at this early stage is needed. This research investigates the relationship between insight and psychotic and depressive symptoms in acute and stable phases of FEP, as well as the association between insight, neuropsychological performance, and social functioning in the stable phase. Moreover, we explore how changes in insight correlate with symptom evolution between the two phases.

**Methods:**

Ninety individuals with FEP were assessed at the acute and/or stable phases of the illness. Insight was assessed using the Scale to Assess Unawareness of Mental Disorder (SUMD) across three dimensions: insight into having a mental disorder (IMD), insight into the effects of medication (IEM), and insight into the social consequences (ISC) of having a mental disorder. Symptoms were assessed with the Positive and Negative Syndrome Scale (PANSS) and Clinical Global Impression-Schizophrenia Scale (CGI-SCH). A battery of cognitive tests was used to assess neurocognition, while social functioning was assessed with the Global Assessment of Functioning Scale (GAF) and the Disability Assessment Schedule (DAS-sv).

**Results:**

During the acute phase, poor insight was significantly correlated with increased positive symptoms (IMD: *p* = 0.002; IEM: *p* = 0.003; ISC: *p* = 0.011) and general symptoms (IMD: *p* = 0.016; IEM *p* = 0.006). In the stable phase, insight remained significantly correlated with positive (IMD: *p* < 0.001; IEM: *p* = 0.010; ISC: *p* = 0.006) and general symptoms (IMD: *p* = 0.003; IEM: *p* = 0.023; ISC: *p* = 0.018). Negative symptoms (IMD: *p =* 0.002; IEM: *p* = 0.004; ISC: *p* = 0.004) and cognitive symptoms (via CGI-SCH) were also correlated with insight (IMD: *p* = 0.010; IEM: *p* = 0.020; ISC: *p* = 0.015). Neuropsychological performance was significantly associated to insight, with executive functioning correlating with IMD (Trail Making Test-A (TMT-A): *p* = 0.002; Trail Making Test-B (TMT-B): *p* = 0.014) and verbal memory correlating with IEM (short-term: *p* = 0.004; long-term: *p* = 0.043). Lower cognitive performance was associated with poorer insight (IMD: *p* = 0.002; IEM: *p* = 0.037; ISC: *p* = 0.008). Improved insight in IMD and ISC was associated with higher psychosocial functioning (GAF: *p* = 0.001; *p* = 0.005) and lower social disability (DAS-sv: *p* = 0.012; *p* = 0.004). Finally, insight improvements correlated with symptom reduction, as a decrease in PANSS positive symptoms was associated with better IMD (*p* < 0.001), while reduced general symptoms correlated with improved IEM (*p* = 0.024). IMD was the only dimension influenced by its acute-phase level (*p* = 0.004).

**Conclusion:**

Understanding the implications of insight in the course and prognosis of psychosis is crucial for achieving positive outcomes. Targeting the three key insight dimensions (insight into illness, medication necessity and social consequences), with tailored interventions adapted to different illness stages can help optimize treatment response.

## Introduction

Poor insight is a prevalent characteristic in psychotic disorders [1]. While the term lacks a single definition, insight is generally considered a multidimensional construct. It is currently conceptualized as a continuum, which evolves and fluctuates within the same individual [2], despite some authors suggesting that insight is trait-dependent and remains stable across the different phases of the disease [3].

Several studies have established a correlation between the level of overall insight and psychotic symptoms, evident in individuals diagnosed with schizophrenia and those experiencing first-episode psychosis (FEP). Most studies have found correlations between impaired insight and greater severity of positive and general symptoms [4, 5, 6]. A smaller number of studies have also found a correlation between poor insight and negative [7, 8] and disorganized symptoms [9], indicating that the precise nature of the association between psychosis and insight remains unresolved. Furthermore, it has been hypothesized that the reduction in psychotic symptoms following an acute episode may be linked to an improvement in the degree of insight [10, 11, 12].

Regarding the interplay between insight and depression, numerous studies suggest a positive correlation between depression and insight, observed both in individuals with schizophrenia [5, 13] and FEP [14]. Nevertheless, other studies have failed to replicate these associations [7, 15, 16].

In addition, researchers have investigated the link between lack of insight and neuropsychological impairments. Numerous studies have examined the relationship between impaired insight and cognitive dysfunction in schizophrenia and FEP [5, 17, 18], while other authors have focused on more concrete areas such as memory alterations [19] and executive functions [18, 20]. Nonetheless, some authors have reported inconclusive results [21]. In terms of the correlation between improved insight and higher intelligence quotient (IQ) in schizophrenia and FEP, various authors have found evidence supporting this relationship [18], while others have not found a significant association [22].

Another relevant line of study concerning insight in psychosis has focused on how it relates to psychosocial functioning. Some studies have revealed a positive correlation between the two and have suggested that the degree may serve as a reliable predictor of the overall level of global functioning in individuals with psychosis, and vice versa [23, 24, 25].

The current study aims to clarify the discrepancies regarding the influence of insight on symptoms, neurocognition, and social functioning, as well as to examine the progression of insight over time and provide some guidance for interventions aimed at improving insight in different clinical settings. Moreover, it is important to assess which variables from the acute and stable phases contribute to insight in the stable phase. The analysis of the differential implications of each of these dimensions could significantly contribute to the study of insight [26].

### Aims

This study aims to: (a) explore the relationship between each insight dimension and clinical symptoms (i.e., psychotic and depressive symptoms), in the acute and stable phases in FEP, (b) examine the correlation between changes in each insight dimension and symptoms from the acute to stable phase, and, (c) evaluate the relationship between each insight dimension and neuropsychological and social functioning in the stable phase, and (d) assess the most relevant variables from the acute and stable phases to understand the role of each of the insight dimensions in the stable phase.

## Methods 

### Sample

The study was carried out between 2007 and 2010.

A total of 90 patients experiencing a FEP were recruited. Adult patients were selected from the acute unit and community services within the mental health services network of Parc Sanitari Sant Joan de Déu, while adolescent patients were recruited from the acute unit of the children’s and adolescents’ mental health services at Hospital Sant Joan de Déu.

To be included in the study, patients had to meet the following inclusion criteria: (1) be between 7 and 65 years old; (2) present at least two psychotic symptoms (such as hallucinations, delusions, disorganized speech, catatonia or disorganized behavior, or negative symptoms); (3) have experienced symptoms for less than a year and had initial contact with medical services within the past 6 months.

Exclusion criteria included: (1) presence of intellectual disabilities (2) history of brain damage, such as traumatic brain injury or other neurological disorders affecting cognitive functioning.

All participants were informed about the study’s objectives and methods by their psychiatrist and provided their written informed consent. For children and adolescents, consent was obtained from both the participants and their parents. The study was approved by the Ethics Committee of Sant Joan de Déu.

### Assessment

Data collection was conducted during the study period (see section below).

Patients were evaluated either during the acute phase of the first episode, the stable phase, or both. The acute phase was defined as the period during which the patient was hospitalized, while the stable phase was assessed approximately two months after discharge.

Insight and clinical symptoms measures were assessed at both time points, during the acute and stable phases. The post-hoc questionnaire was administered in the acute phase, while neuropsychological and psychosocial functioning assessments were conducted in the stable phase.

Sociodemographic variables, including gender, marital status, housing situation, age, educational level, and occupational status, such as age at onset and duration of untreated psychosis (DUP), were collected using a post-hoc questionnaire.

#### Insight

The Scale to Assess Unawareness of Mental Disorder (SUMD) [27, 28] is a semistructured interview that evaluates insight in mental disorders across three dimensions: (a) insight about having a mental disorder (IMD), (b) insight into the effects of medication (IEM), and (c) insight into the social consequences of having a mental disorder (ISC). Each dimension is scored from 1 to 5, with 1 indicating good insight, 3 indicating partial insight, and 5 indicating poor insight [27]. Thus, higher scores represent worse insight.

#### Clinical Symptoms

The Positive and Negative Syndrome Scale (PANSS) [29, 30] was used to evaluate psychotic symptoms. This scale assesses 30 symptoms on a scale from 1 (absence of symptoms) to 7 (extreme symptom severity), with higher scores indicating more severe psychopathology. The PANSS is composed of three subscales: positive (7 items), negative (7 items), and general (16 items) symptoms. Although this scale was originality designed for adults, it is a widely accepted outcome measure in clinical trials for pediatric schizophrenia [31].

The Clinical Global Impression-Schizophrenia Scale (CGI-SCH) was used to assess the severity of the illness. This scale includes four subscales: positive symptoms, negative symptoms, depressive symptoms, and cognitive symptoms, along with the overall severity of the mental disorder. Each subscale is rated from 1 (normal, not ill) to 7 (most severe illness). Higher scores on the CGI-SCH indicate greater severity of the illness [32].

#### Neurocognition

Neurocognition was evaluated using a series of tests.

The Continuous Performance Test (CPT) assessed inhibition, sustained attention, and processing speed, with variables including errors of omission and commission [33]. Higher error rates indicate greater attentional deficits and impaired cognitive control.

The Trail Making Test-A (TMT-A) and Trail Making Test-B (TMT-B) measured attention, cognitive flexibility, and motor speed [34]. TMT-A requires connecting numbers sequentially, while TMT-B involves alternating between numbers and letters. Longer completion times indicate poorer executive functioning and processing speed.

The Stroop Test evaluated selective attention, inhibition, and the speed of automatic cognitive processes [35]. Longer reaction times and higher error rates indicate greater difficulties in cognitive control and response inhibition.

The Wechsler Adult Intelligence Scale (WAIS) for individuals over 15 years old and the Wechsler Intelligence Scale for Children (WISC) for those under 16 years old [36, 37] were used to estimate IQ. This estimation was based on the vocabulary subtest, as recommended by previous research [38]. Higher scores reflect greater verbal intellectual ability.

The Verbal Learning Test España-Complutense (TAVEC) for individuals over 15 years old and the Verbal Learning Test España-Complutense Children (TAVECI) for those under 16 years old [39], both of which are similar to the California Verbal Learning Test, evaluated memory. The analysis included variables such as working memory (WM), short-term memory (SM), and long-term memory (LM). Additionally, the type of strategy used for word recall was evaluated, distinguishing between serial recall (following the order of presentation) or semantic recall (grouping words by category) for both SM and LM. The number of intrusions (recalling non-existent words) was also recorded. T-scores were used, as these are adjusted for demographic characteristics, with higher scores indicating better memory performance.

#### Psychosocial Functioning

The Global Assessment of Functioning Scale (GAF) was used to measure overall functioning on a scale from psychiatric illness to health, with a score of 1 indicating severe dysfunction and 100 indicating optimal functioning [40]. Lower scores indicate greater functional impairment.

The Disability Assessment Schedule-short version (DAS-sv) [41, 42] assessed psychosocial functioning. This scale provides a total score of disability assessment (ranging from 0 to 20) and individual scores in areas such as personal care, occupational disability, family disability, and social disability (each ranging from 0 to 5). Higher scores indicate greater disability.

### Statistical Analysis

Data were analyzed using SPSS v.24 (IBM Corp., Armonk, NY, USA). Descriptive statistics were used to summarize the variables. Data normality was assessed using the Shapiro-Wilk test and by visually inspecting histograms and Q-Q plots. Depending on the data distribution, either parametric or non-parametric statistical tests were applied.

The degree of insight was compared between the two time points using the Chi-square test. The relationship between insight and clinical or neuropsychological variables was examined using Pearson’s correlation for normally distributed variables and Spearman’s correlation for non-normally distributed variables. To investigate the variables most closely associated with insight in the stable phase, a multiple regression (stepwise method) was conducted, including all significant variables from the bivariate analysis. Given the exploratory nature of this study, we did not apply multiple comparison corrections [43].

## Results

A total of 90 patients experiencing a FEP were recruited. Of these, 71 patients were recruited from an acute inpatient unit and 19 from community mental health services within the Sant Joan Déu network.

Table 1 shows the sociodemographic variables of the sample. It was comprised of 57.8% men and 42.2% women, aged between 13 and 47 years, with a mean age of 20.62. About 85.5% were living with their parents and 88.9% were single. A total of 51.1% were students, while 33.3% were employed. The average length of time between baseline assessment in the acute services and follow-up in the outpatient care was 68.35 days (standard deviation (SD) = 73.71).

**Table 1.** **Sociodemographic variables of the sample**.

		N	%
Gender	Male	52	57.8
	Female	38	42.2
Living with	Alone	3	3.3
	Couple/partner	2	2.2
	Parents	77	85.5
	Own family	4	4.4
	Others	4	4.4
Marital status	Single	80	88.9
	Married	7	7.8
	Divorced	3	3.3
Level of education	Primary	51	56.7
	Secondary	24	26.6
	University	15	16.7
Occupational status	Employed	30	33.3
	Student	46	51.1
	Sick leave	8	8.8
	Other situations	6	6.6
		Mean (SD)	Range
Age		20.62 (6.92)	13–47
Age at onset		20.58 (7.01)	13–47
DUP (number of days)		97 (136.11)	0–708

DUP, duration of untreated psychosis; SD, standard deviation.

Although 90 participants were recruited, complete data were not available for all individuals across both assessment phases and insight dimensions due to missing responses in certain SUMD items. As a result, the number of valid cases varied by dimension and phase. Table 2 displays the frequency and percentage distribution of insight levels for each SUMD dimensions across assessments, which served as the basis for subsequent analyses examining changes in insight over time.

**Table 2.** **Frequencies and percentages of insight degree for each SUMD dimensions in acute and stable phases**.

		Acute phase		Stable phase	
		(*n* = 74¹/71²/73³)		(*n* = 81¹/78²/81³)	
		N	%	N	%
IMD^1^	Good	11	14.9%	39	48.1%
	Partial	17	23%	19	23.5%
	Poor	46	62.1%	23	28.4%
IEM^2^	Good	37	52.1%	60	76.9%
	Partial	14	19.7%	11	14.1%
	Poor	20	28.2%	7	9%
ISC^3^	Good	22	30.1%	49	60.4%
	Partial	12	16.4%	16	19.8%
	Poor	39	53.5%	16	19.8%

IMD, insight of mental disorder; IEM, insight of the effects of medication; ISC, insight of social consequences of a mental disorder. Insight levels were categorized based on SUMD scores as follows: good insight = scores 1–2; partial insight = score 3; poor insight = scores 4–5. Percentages reflect valid responses per dimension and phase. Slight variations in total n across phases are due to missing data in specific items. ^1^*n* for IMD: acute phase = 74, stable phase = 81. ^2^*n* for IEM: acute phase = 71, stable phase = 78. ^3^*n* for ISC: acute phase = 73, stable phase = 81.

The mean insight score in the acute phase was 3.95 (SD = 1.49) for IMD, 2.52 (SD = 1.74) for IEM, and 3.47 (SD = 1.78) for ISC. In the stable phase, the scores were lower, with a mean of 2.60 (SD = 1.72) for IMD, 1.64 (SD = 1.27) for IEM, and 2.19 (SD = 1.61) for ISC.

Chi-square tests were conducted to examine a significant change in the distribution of insight levels between the acute and stable phases. These analyses were based on the subsample of participants who completed all three insight dimensions at both time points, specifically 65 participants for IMD, 60 for IEM, and 64 for ISC. A significant change was observed for the IMD dimension (χ^2^ = 11.88, *p* = 0.018), but no for the other dimensions (IEM: χ^2^ = 5.94, *p* = 0.203; ISC: χ^2^ = 6.23, *p* = 0.182).

### Correlation Between Insight and Clinical Symptoms in Both Acute and Stable Phases 

Two sets of correlation analyses were conducted: the first examining the relationship between clinical variables and insight during the acute phase, and the second analyzing these correlations in the stable phase.

The findings revealed that poor insight (higher SUMD scores) during the acute phase was associated with heightened positive symptoms, as assessed across all three subscales of the SUMD and the PANSS (Table 3). Significant correlations were also found between positive symptoms measured by the CGI-SCH and insight dimensions IMD and IEM. Moreover, general symptoms assessed with the PANSS also showed significant correlations with the same dimensions of insight (IMD and IEM). Notably, PANSS item G12 (insight) was significantly correlated with IMD (*p* < 0.001), IEM (*p* = 0.005) and ISC (*p* < 0.001) during the acute phase.

**Table 3.** **Relationship between insight and clinical symptoms in the acute and stable phases**.

			Acute phase			Stable phase		
			IMD	IEM	ISC	IMD	IEM	ISC
PANSS	Positive	Correlation	0.357**	0.350**	0.295*	0.383***	0.290*	0.302**
		*p* value	0.002	0.003	0.011	<0.001	0.010	0.006
	Negative	Correlation	0.121	0.044	–0.017	0.341**	0.324**	0.314**
		*p* value	0.310	0.718	0.888	0.002	0.004	0.004
	General	Correlation	0.279*	0.326**	0.108	0.330**	0.258*	0.262*
		*p* value	0.016	0.006	0.364	0.003	0.023	0.018
CGI-SCH	Positive	Correlation	0.240*	0.254*	0.151	0.429***	0.215	0.317**
		*p* value	0.039	0.032	0.202	<0.001	0.059	0.004
	Negative	Correlation	0.188	0.080	0.018	0.342**	0.286*	0.372**
		*p* value	0.108	0.509	0.877	0.002	0.011	0.001
	Depressive	Correlation	–0.018	–0.004	–0.223	0.006	0.055	0.061
		*p* value	0.881	0.976	0.058	0.959	0.630	0.588
	Cognitive	Correlation	0.001	–0.046	–0.051	0.283*	0.262*	0.270*
		*p* value	0.993	0.705	0.669	0.010	0.020	0.015
	Total	Correlation	0.263*	0.273*	0.100	0.422***	0.231*	0.430***
		*p* value	0.023	0.021	0.402	<0.001	0.042	<0.001

IMD, insight of mental disorder; IEM, insight of the effects of medication; ISC, insight of social consequences of a mental disorder; PANSS, Positive and Negative Syndrome Scale; CGI-SCH, Clinical Global Impression-Schizophrenia Scale. PANSS and CGI-SCH dimensions are correlated with each domain of insight considering acute or stable phase according with the information of the insight domains. *p < 0.05. **p < 0.01. ***p < 0.001.

In the stable phase, both positive and negative symptoms, as assessed using the PANSS and the CGI-SCH, showed significant correlations with all three SUMD dimensions, except for the correlation between CGI-SCH positive symptoms and IEM (Table 3), which was not significant. Furthermore, cognitive symptoms evaluated with the CGI-SCH and general symptoms from the PANSS subscale were also correlated with all three SUMD insight dimensions (Table 3). Similarly, PANSS item G12 (insight) remained significantly correlated with IMD (*p* < 0.001), IEM (*p* < 0.001) and ISC (*p* < 0.001) in the stable phase.

No significant correlation was found between depressive symptoms (CGI-SCH) and any SUMD insight dimension in either phase.

### Changes in Insight and Symptoms Between the Acute and Stable Phases

Regarding changes in insight from the acute phase to the stable phase, high percentages of insight maintenance and improvement were observed, with only a small proportion of patients showing a decline. Specifically, for IMD, 12% of the sample showed good insight in the acute phase and maintained the level in the stable phase, while the figures were 27% for IEM and ISC. Similarly, 57% of patients showed improvement in their insight levels in the change of phase for IMD, 27% for IEM, and 43% for ISC. Patients with poor insight in the acute phase had the tendency to remain at the same level at the stable phase, with no significant change in insight across the three dimensions: 22% for IMD subscale, 20% for IEM and 13% for ISC.

To determine whether the symptoms improvement were associated with increased insight, changes in PANSS scores between the acute and stable phases were compared with SUMD scores. The results indicated that a reduction in positive symptoms correlated with improvement in IMD, while a reduction in general symptoms correlated with improvement in IEM (Table 4).

**Table 4.** **Correlations between changes in insight and symptoms between the acute and stable phases**.

		Improvements in IMD	Improvements in IEM	Improvements in ISC
Improvements in PANSS Positive	Correlation	0.453***	0.167	0.240
	*p* value	<0.001	0.203	0.056
Improvements in PANSS Negative	Correlation	0.052	0.021	–0.175
	*p* value	0.684	0.876	0.171
Improvements in PANSS General	Correlation	0.174	0.291*	–0.099
	*p* value	0.166	0.024	0.438

IMD, insight of mental disorder; IEM, insight of the effects of medication; ISC, insight of social consequences of a mental disorder; PANSS, Positive and Negative Syndrome Scale. *p < 0.05. ***p < 0.001.

### Correlations Between Insight and Neuropsychological Performance

In the cognitive assessment, significant correlations were found between neuropsychological performance and several SUMD insight dimensions (Table 5). Specifically, errors of omission on the CPT correlated with ISC. Poorer performance on TMT-A and TMT-B correlated with lower IMD insight. Regarding verbal memory, a lower level of IEM correlated with the use of a serial recall strategy in both short-term and long-term memory, while a lower level of ISC with a higher number of intrusions. Finally, a negative correlation was identified between estimated IQ and all dimensions of insight.

**Table 5.** **Correlations between insight and neuropsychological performance**.

			IMD	IMD	ISC
CPT	Errors of omission	Correlation	0.189	0.038	0.288*
		*p* value	0.113	0.757	0.014
	Errors of commission	Correlation	0.134	0.105	–0.027
		*p* value	0.261	0.390	0.819
Stroop		Correlation	–0.212	–0.083	–0.068
		*p* value	0.064	0.483	0.556
TMT	A	Correlation	0.342**	0.148	0.208
		*p* value	0.002	0.210	0.069
	B	Correlation	0.279*	0.177	0.134
		*p* value	0.014	0.131	0.247
TAVEC	WM	Correlation	–0.031	–0.223	–0.078
		*p* value	0.795	0.057	0.508
	SM	Correlation	0.200	0.198	0.179
		*p* value	0.086	0.093	0.124
	LM	Correlation	0.027	0.196	–0.032
		*p* value	0.815	0.096	0.785
	Serial strategy	Correlation	0.076	0.336**	0.148
	SM	*p* value	0.517	0.004	0.206
	Serial strategy	Correlation	–0.034	0.238*	–0.031
	LM	*p* value	0.772	0.043	0.794
	Semantic strategy	Correlation	–0.219	0.015	–0.143
	SM	*p* value	0.059	0.899	0.220
	Semantic strategy	Correlation	–0.071	0.115	–0.027
	LM	*p* value	0.543	0.332	0.818
	Intrusions	Correlation	0.123	–0.127	0.230*
		*p* value	0.297	0.289	0.049
Estimated IQ		Correlation	–0.351**	–0.246*	–0.303**
		*p* value	0.002	0.037	0.008

IMD, insight of mental disorder; IEM, insight of the effects of medication; ISC, insight of social consequences of a mental disorder; TAVEC, Verbal Learning Test España-Complutense; TMT, Trail Making Test; CPT, Continuous Performance Test; WM, Working memory; SM, Short-term memory; LM, Long-term memory; IQ, Intelligence Quotient. *p < 0.05. **p < 0.01.

### Correlations Between Insight and Psychosocial Functioning 

Concerning psychosocial functioning, higher overall functioning, as measured by the GAF, was significantly correlated with lower levels of IMD and ISC (Table 6). Additionally, only the social disability subscale of the DAS-sv was correlated with the same insight dimensions (IMD and ISC).

**Table 6.** **Correlations between insight and psychosocial functioning**.

			IMD	IEM	ISC
GAF		Correlation	–0.352**	–0.175	–0.312**
		*p* value	0.001	0.170	0.005
DAS-sv	Personal care	Correlation	0.173	0.014	0.291*
		*p* value	0.134	0.908	0.011
	Occupational disability	Correlation	0.072	0.026	0.070
		*p* value	0.542	0.830	0.555
	Family disability	Correlation	0.132	–0.076	0.071
		*p* value	0.258	0.525	0.547
	Social disability	Correlation	0.298*	0.072	0.325**
		*p* value	0.012	0.549	0.004

IMD, insight of mental disorder; IEM, insight of the effects of medication; ISC, insight of social consequences of a mental disorder; GAF, Global Assessment of Functioning scale; DAS-sv, Disability Assessment Schedule-short version. *p < 0.05. **p < 0.01.

### Predicting Factors of Insight in the Stable Phase

Table 7 shows the factors that predicted each insight dimension in the stable phase. IMD in the stable phase was influenced by CGI-SCH positive symptoms in the stable phase, IMD in the acute phase, and IQ. Similarly, IEM in the stable phase was associated with the use of a serial recall strategy in verbal memory, PANSS positive symptoms in the stable phase, and IQ. Lastly, ISC in the stable phase was predicted by CGI-SCH positive symptoms in the stable phase, CPT omissions, and IQ.

**Table 7.** **Regression model of insight dimensions in the stable phase**.

	Stable Phase					
	IMD		IEM		ISC	
Variables in the model	B	*p* value	B	*p* value	B	*p* value
Positive symptoms CGI stable phase	0.420	0.003			0.513	<0.001
Positive symptoms PANSS stable phase			0.063	0.043		
IMD acute phase	0.396	0.004				
IQ	–0.030	0.008	–0.027	0.010	–0.045	<0.001
Serial strategy short term			0.058	0.004		
Omissions CPT					0.016	0.048
R2	0.333		0.253		0.452	

IMD, insight of mental disorder; IEM, insight of the effects of medication; ISC, insight of social consequences of a mental disorder; CPT, Continuous Performance Test; IQ, Intelligence Quotient; PANSS, Positive and Negative Syndrome Scale; CGI-SCH, Clinical Global Impression-Schizophrenia Scale; R2, R-squared.

Except for IMD, no other variable in the acute phase was significant in the predictive models.

## Discussion

Our findings imply that, both during the acute and stable phases of FEP, psychotic symptoms and clinical insight are significantly related. Furthermore, as patients transition from the acute to the stable phase, improvements in insight correspond to insight reduction. Some neuropsychological variables and social functioning measures are related to specific insight dimensions. Finally, regression analysis shows that positive symptoms, IQ, and specific neuropsychological variables, such as memory and attention, play a role in explaining insight dimensions in the stable phase.

The mean scores for each of the three scales of the SUMD were comparable to those reported in other studies with FEP samples [44]. Regarding the distribution of patients by degree of insight, our results align with findings from previous FEP samples [45, 46], which report a higher proportion of individuals with good or partial insight compared to studies on early-phase schizophrenia [47].

A greater degree of insight correlated with lower scores in positive and general symptoms in the acute phase and with positive, negative, and general symptoms in the stable phase. These findings are consistent with previous research on FEP, both in cross-sectional studies of inpatients [48, 49] and longitudinal studies assessing stable phases [5, 50]. More specifically, a regression analysis revealed that positive symptoms in the stable phase contributed to insight in that phase. The absence of a correlation between insight and negative symptoms in the acute phase aligns with findings from previous studies on FEP [50, 51]. Interestingly, while no association was observed between general symptomatology and the insight of social consequences of having a mental disorder in the acute phase, this correlation emerged in the stable phase, indicating that awareness of social consequences develops as patients reintegrate into the community.

We also found a correlation between all three dimensions of the SUMD and cognitive symptoms (CGI-SCH) in the stable phase. This result is consistent with previous studies that have linked insight with disorganization symptoms, as measured by the PANSS, in FEP [52]. The CGI-SCH scale is similar to the disorganization dimension of the five-factor PANSS scale [33].

Regarding depressive symptoms, no correlation was found. However, some studies involving FEP patients have reported a relationship between depressive symptoms and insight [24, 53], while others failed to replicate these findings [14, 54], resulting in inconclusive evidence. A possible explanation for our results is the psychological model proposed by [55], which suggests that depression emerges as part of a cognitive recovery process following an acute episode, in line with previous findings [49].

Regarding the predictive model, only one variable in the acute phase was significant in the models: the IMD score in the acute phase contributed to predicting IMD levels in the stable phase. These results contribute to the discussion on whether the lack of insight is an intrinsic feature of the disorder itself [56]. In this line, IMD could be more stable over time compared to IEM and ISC [2].

Our results show that, as patients transition from the acute phase to the stable phase, their level of insight improves, suggesting a correlation between increased insight and symptom reduction. These results agree with the existing literature [25, 57]. Furthermore, reductions in positive and general symptoms were associated with improvements in IMD and IEM, respectively, supporting the initial hypothesis that lack of insight is a defining characteristic of the acute phase [56]. Additionally, the correlation between general symptom reduction and IEM improvement may be related to the established link between poor treatment adherence and worse overall illness outcomes [58]. Several studies have reached similar conclusions, linking clinical recovery with improved insight scores in FEP [16].

Concerning cognitive performance, only TMT-A and TMT-B correlated with IMD, while omission errors in the CPT were linked to ISC. Omission errors in the CPT were also significant in the regression model for ISC. Although a trend was found between Stroop performance and IMD, it did not reach statistical significance. The association between poor insight and deficits in executive functions in FEP samples is consistent across studies [19, 59], while other publications have failed to find this association [60]. Our results support previous findings indicating that attention deficits may hinder information processing and integration, leading to systematic errors in self-assessment regarding mental disorders [61].

In terms of verbal memory, our analysis did not reveal an association between insight and WM, SM, and LM variables, partially aligning with the findings reported in a previous meta-analysis [18]. However, a noteworthy finding in our study was that individuals with poor IEM used a serial memorization strategy. In fact, the use of serial strategy was one of the predictors of IEM dimension of insight in the regression model. This observation is consistent with cognitive models of information, which propose that patients with schizophrenia struggle to organize information, resulting in errors in stimulus association [62, 63]. Along this line, some studies have described how patients with schizophrenia show increased confidence in memory errors, often misinterpreting new information as external, which may negatively impact social interactions [64].

In summary, as previously suggested [49] the association between cognitive impairment and lack of insight in FEP remains unclear, and poor insight may be more closely related to metacognitive deficits than to cognitive impairment per se.

As expected [18, 19], IQ emerged as a significant predictor, with higher estimated IQ correlating with greater degree of insight across all the three SUMD dimensions. In addition, IQ was also the single significant variable that contributed to all insight dimensions in the regression model.

Finally, in the line with previous research [61, 65], poor overall psychosocial and social functioning was associated with poor IMD and ISC scores.

This study has limitations, notably the potential for greater illness severity in our sample of FEP patients, limited to those requiring hospital admission. Discrepancies with other studies may stem from sample size variations, differences in depression measurement scales, neurocognitive assessment tools, and psychosocial functioning scales. Another possible limitation is the assumption that adolescents could assess insight in the same way as adults; however, in early adolescence, self-awareness begins to develop more strongly, along with other capacities such as self-consciousness and self-reflection [66, 67]. Thus, as these cognitive abilities continue to mature into early adulthood, SUMD scores in younger participants may reflect both illness-related impairments and normative developmental differences [68]. Future research should explore how brain maturation and cognitive flexibility influence insight in early psychosis. Additionally, the broad age range included in this study may introduce additional variability in insight assessment, as neurodevelopmental and cognitive factors differ significantly between adolescents and adults. To better capture these differences, future studies should aim to expand the sample size and analyze these age groups separately. Moreover, the variation in sample size across insight dimensions and assessment phases reflects the longitudinal design of the broader GENIPE study and the presence of missing data. Some participants recruited during hospitalization were lost to follow-up or provided incomplete responses, while others recruited through community services were only assessed during the stable phase. This highlights the challenges of conducting longitudinal research with real-world clinical populations. Another limitation is the absence of a healthy control group, which limits the ability to determine whether the observed relationships between insight and psychotic symptoms are specific to the disorder. Including a control group in future research would help disentangle disease-specific patterns from general cognitive processes. Lastly, exploration of insight correlations with sociodemographic variables is lacking. Future research should address this gap, examining the impact of variables like gender on follow-up and the interplay between symptoms and insight, as suggested by previous studies [69].

Our study includes a multidimensional perspective on insight, revealing distinct correlations of each dimension with symptoms, neuropsychological and social functioning. This approach is underrepresented in FEP research, highlighting the need for further investigation in this area. Based on our findings, tailored interventions should target IMD and IEM during early illness phases, from the acute phase through symptom remission. Both dimensions are strongly linked to non-adherence and poorer outcomes from the onset of psychosis [2]. In the stable phase, addressing insight into ISC becomes helpful as patients reintegrate into the community post-discharge. While evidence supports the efficacy of treatment and intervention designed to increase insight, further research is needed [26, 70].

## Conclusion

In conclusion, insight is a key factor in the clinical course of FEP, showing its associations with symptomatology, neurocognition, and psychosocial functioning. Improvements in insight over time were linked to reductions in positive and general symptoms, reinforcing the dynamic nature of insight during recovery. Given the differential implications of each insight dimension, targeted interventions should address insight into illness, medication necessity, and social consequences at different illness stages. Understanding these mechanisms can help optimize treatment strategies and improve long-term outcomes for individuals with early psychosis.

## Availability of Data and Materials

Due to confidentiality issues, access to data will only be granted on request (SO; susana.ochoa@sjd.es; JU; judith.usall@sjd.es).

## Explanation of Special Symbols

§ Member of the GENIPE Group (Grupo de Investigación en Epidemiología Psiquiátrica).
